# Chemical Activation of the Hypoxia-Inducible Factor Reversibly Reduces Tendon Stem Cell Proliferation, Inhibits Their Differentiation, and Maintains Cell Undifferentiation

**DOI:** 10.1155/2018/9468085

**Published:** 2018-03-11

**Authors:** Alessandra Menon, Pasquale Creo, Marco Piccoli, Sonia Bergante, Erika Conforti, Giuseppe Banfi, Pietro Randelli, Luigi Anastasia

**Affiliations:** ^1^IRCCS Policlinico San Donato, San Donato Milanese, Milan, Italy; ^2^IRCCS Istituto Ortopedico Galeazzi, Milan, Italy; ^3^Università Vita-Salute San Raffaele, Milan, Italy; ^4^Azienda Socio Sanitaria Territoriale Centro Specialistico Ortopedico Traumatologico Gaetano Pini-CTO, Milan, Italy; ^5^Department of Biomedical Sciences for Health, University of Milan, Milan, Italy

## Abstract

Adult stem cell-based therapeutic approaches for tissue regeneration have been proposed for several years. However, adult stem cells are usually limited in number and difficult to be expanded in vitro, and they usually tend to quickly lose their potency with passages, as they differentiate and become senescent. Culturing stem cells under reduced oxygen tensions (below 21%) has been proposed as a tool to increase cell proliferation, but many studies reported opposite effects. In particular, cell response to hypoxia seems to be very stem cell type specific. Nonetheless, it is clear that a major role in this process is played by the hypoxia inducible factor (HIF), the master regulator of cell response to oxygen deprivation, which affects cell metabolism and differentiation. Herein, we report that a chemical activation of HIF in human tendon stem cells reduces their proliferation and inhibits their differentiation in a reversible and dose-dependent manner. These results support the notion that hypoxia, by activating HIF, plays a crucial role in preserving stem cells in an undifferentiated state in the “hypoxic niches” present in the tissue in which they reside before migrating in more oxygenated areas to heal a damaged tissue.

## 1. Introduction

**Figure 1:**
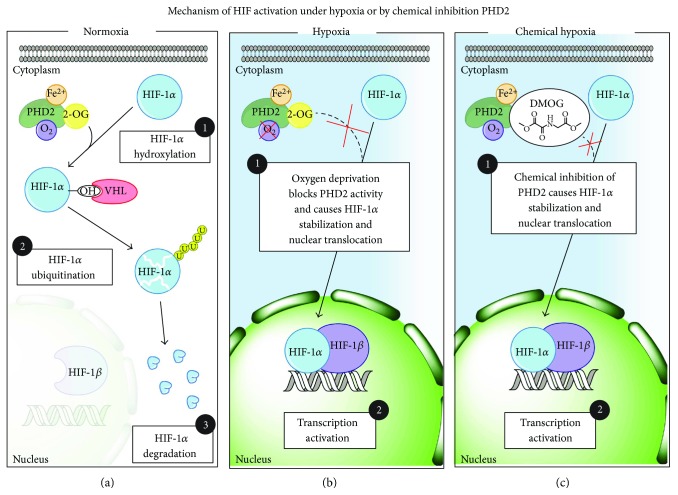
Schematic representation of different mechanisms of HIF-1*α* activation. The picture shows a schematic representation of (a) HIF-1*α* degradation by proteasome under normoxic conditions, (b) HIF-1*α* stabilization under hypoxic conditions mediated by the oxygen deprivation which causes the inhibition of PHD2 activity, and (c) chemical-induced HIF-1*α* stabilization under normoxic conditions by the inhibition of PHD2 activity through DMOG treatment.

Stem cell-based therapies have promised to be an attractive approach in tissue regeneration, especially with the discovery that adult tissues possess a reservoir of progenitor cells that can be isolated and cultured in vitro [1]. However, one of the main obstacles that have been encountered is the relatively small number of adult stem cells present in tissues and their very limited proliferative capacity. Moreover, while stem cells can be easily isolated from patients, they usually tend to quickly lose their potency in vitro with passages, as they differentiate and become senescent. A possible approach to overcome these limitations is to culture cells under low oxygen levels (below 21%) [2–5]. However, maintaining the correct in vitro culturing conditions under reduced oxygen tensions is quite complex. In fact, it requires special hypoxic chambers to avoid subjecting cells to continuous hypoxia/reoxygenation cycles during medium changes. Moreover, results are often conflicting, as many authors reported both the inhibition and promotion of cell proliferation even under moderate hypoxia (5–10%) [6–9]. Clearly, one confounding element is that adult stem cells are a very heterogeneous class of different cell subtypes, which are tissue specific. In addition, isolation techniques and culturing conditions in vitro heavily affect their phenotype. Therefore, we can speculate that these inconsistencies in the literature are likely due to a distinct response of each stem cell subtype to hypoxia. Nonetheless, under reduced oxygen tensions, it is well established that the alpha subunit of the hypoxia inducible factor (HIF-1*α*) is stabilized [10], and this causes a signaling cascade greatly affecting stem cell metabolism and differentiation [11–14] (Figure 1(a)). One interesting feature of HIF mechanism of activation is that its stabilization relies on the inhibition of a family of prolyl hydroxylases (PHDs) that require oxygen to work; thus, their activity is reduced under oxygen starvation (Figure 1(b)). Interestingly, it has been shown that a chemical inhibition of PHD activity, using for instance 2-oxoglutarate analogous like dimethyloxalylglycine (DMOG), can mimic the effects of hypoxia in activating HIF even under normal oxygen tension (Figure 1(c)). Therefore, it could be envisioned to add these inhibitors in the culture medium to finely modulate stem cell proliferation and differentiation, avoiding the complex control of the oxygen tension.

On these bases, in this work, we studied the effects of HIF-1*α* stabilization, obtained by chemically inhibiting the PHDs, on human tendon stem cells, that we recently isolated for the first time from the human rotator cuff tendons [15, 16].

## 2. Methods

### 2.1. Cell Isolation and Culture

Human tendon stem cells (hTSCs) were isolated from supraspinatus tendon specimens collected during arthroscopic rotator cuff repair, according to a previously reported procedure [15]. The protocol study was approved by the Hospital Ethical Committee with authorization number 2642 (Sept. 19, 2011). Samples from supraspinatus tendons (4–8 mm wide) were collected from 6 patients after signed informed consent, kept in HypoThermosol (BioLife Solutions) at 4°C, and processed separately within 24 h, according to the procedure described below. Samples were washed with phosphate-buffered saline (PBS) (Euroclone), cut into small pieces, and digested for 90 min with collagenase type I (3 mg/mL; Worthington) and dispase (4 mg/mL; Gibco, Life Technologies) in PBS at 37°C. After centrifugation, cell pellets were resuspended in the following culture medium: *α*-Minimal Essential Medium (*α*-MEM) (Sigma-Aldrich) supplemented with 2 mM glutamine (Euroclone), 1% antibiotic-antimycotic mixture (Euroclone), and 20% (*v/v*) fetal bovine serum (FBS) (HyClone, Thermo Fisher Scientific). Cells were then filtered with a cell strainer (70 mM; BD Falcon) and plated in 150 cm^2^ dishes. Adherent cells were cultured at 37°C in a humidified atmosphere of 5% CO_2_. The medium was changed every 2-3 days. All experiments were carried out with cells at passage four to six after isolation [17].

### 2.2. Flow Cytometric Analysis

hTSCs were detached and collected in PBS at a concentration of 2 × 10^6^ cells/mL. Specific binding sites were blocked with a blocking solution (50% 1X PSB, 50% FBS) for 30 minutes at room temperature (RT). Cells were stained with antibodies against human: PE-conjugated CD105, PE-conjugated CD90, FITC-conjugated CD73, conjugated CD45, PerCP-eFluor 710-conjugated CD34, and FITC-conjugated HLA-DR, for 10 minutes at 4°C. The respective isotype antibodies were used as controls. Cell samples were analyzed with a Navios flow cytometer (Beckman Coulter) equipped with Kaluza software (Beckman Coulter).

### 2.3. Chemical Activation of HIF-1*α* with DMOG

DMOG was prepared by solubilization in H_2_O, as suggested by the manufacturer's protocol (Sigma Aldrich). Twenty-four hours after seeding, cells were either cultured for 96 h in normal growth medium with different concentrations of DMOG (0.01 mM, 0.1 mM, and 1 mM) or in normal growth medium without DMOG (controls). The medium was changed every 48 h in all experiments unless specified. To study the reversibility of the effects of DMOG on cell viability and proliferation capacity, multilineage differentiation potential of hTSCs was tested upon DMOG removal from the culture medium. To this purpose, cells were first cultured in the presence of different concentrations of DMOG for 96 h, as described above, washed twice with PBS, detached, and then reseeded and grown in normal medium without DMOG.

### 2.4. Western Blot Analysis

Control hTSCs or treated with different concentrations of DMOG for 96 h were rinsed twice with cold PBS, harvested, and lysed in RIPA buffer containing protease and phosphatase inhibitors. Protein concentrations were measured by the Pierce™ BCA Protein Assay Kit (Thermo Fisher Scientific), following the manufacturer's protocol. Proteins (30 *μ*g) were denatured by boiling for 5 min in sample buffer (6.5 mM Tris-HCl, pH 6.8, 2% SDS (*w/v*), 10% glycerol (*v/v*), 5% 2-mercaptoethanol (*w/v*), and 0.01% bromophenol blue (*w/v*)) and separated on a 10% polyacrylamide gel in denaturing conditions. Proteins were subsequently transferred into nitrocellulose membranes by electroblotting using 100 volts for 2 hours. Then, the membranes were incubated overnight in Tris-buffered saline (TBS: 10 mM Tris-HCl, pH 7.4, 150 mM NaCl), 0.1% (*v/v*) Tween 20 (TBS-Tween) containing 5% (*w/v*) dried milk or 5% (*w/v*) bovine serum albumin (BSA; Sigma-Aldrich) for the blocking buffer. Blots were incubated with a primary antibody specific for HIF-1*α* (Cell Signaling Technology) diluted 1 : 1000 in the appropriate blocking solution for three hours at room temperature. Membranes were then washed four times for 10 min with TBS-Tween and then incubated with the appropriate secondary antibody horseradish peroxidase (HRP) conjugated for 1 hour at room temperature. After four washes in TBS-Tween, the protein bands were detected using an ECL detection kit (Thermo Fisher Scientific) as described by the manufacturer. Relative protein expression levels were calculated normalizing data on Lamin A/C, which was used as internal control.

### 2.5. Cell Morphology and Proliferation

Cells were plated at a concentration of 2.6 × 10^3^ cells/cm^2^ and cultured in normal growth medium in the presence of DMOG (0 mM, 0.01 mM, 0.1 mM, and 1 mM), as described above. Cell morphology was examined with a phase-contrast microscope (Axiovert 40 CFL, Zeiss, equipped with a Moticam 2300 camera, Motic) at each time point to assess the effects of DMOG treatment on hTSC phenotype. Cell growth curves were prepared after harvesting with Trypsin-EDTA solution (Sigma-Aldrich) by counting cells with a Countess Cell Counter (Invitrogen, Life Technologies), according to the manufacturer's procedure. Cell viability was determined by trypan blue dye exclusion assay. All assays were carried out in triplicates for each sample.

### 2.6. Cell Viability by MTT Assay

hTSCs were plated in 12-well plates (1 × 10^4^ cells/well) and treated with different concentrations of DMOG or cultured in the growth medium alone. At each time point (0, 24, 48, and 72 h), two hours before collection, the reconstituted 3-(4,5-dimethylthiazol-2-yl)-2,5-diphenyl-2H-tetrazolium bromide (MTT) (5 mg/mL in PBS; Sigma-Aldrich) was added to the medium (10% of the final volume). Following a two-hour incubation at 37°C, cells were lysed by adding an amount of MTT solubilization solution equal to the original culture medium volume, gently pipetting to completely dissolve the MTT formazan crystals. The MTT reduction was spectrophotometrically measured at a wavelength of 570 nm.

### 2.7. Cell Viability Assay by RealTime-Glo™

Cell viability was assessed using RealTime-Glo MT Cell Viability Assay (Promega) after 48 and 96 h of DMOG treatment (0 mM, 0.01 mM, 0.1 mM, and 1 mM) according to the manufacturer's protocol. The luminescent signal was measured at 48 and 96 h using VICTOR X3™ Multilabel Plate Reader. Briefly, MT cell viability substrate and NanoLuc® enzyme were both diluted 1 : 1000 in culture medium at a final volume of 100 *μ*L per well. Cells were seeded at a density of 500 cells/well in a 96-well plate. Data were normalized to the untreated control.

### 2.8. Cytotoxicity Assay by CellTox Method

CellTox™ cytotoxicity assay (Promega) was used to investigate the cytotoxic effect of DMOG treatment, as the assay measures changes in membrane integrity that occur as a result of cell death. Briefly, cells were seeded in a 96-well plate at a concentration of 500 cells/well and incubated for 48 and 96 h with DMOG (0 mM, 0.01 mM, 0.1 mM, and 1 mM) and tested according to manufacturer's protocol. After each incubation, an equal volume of 100 *μ*L of CellTox buffer containing 1 : 500 dilution CellTox Green Dye was added to each well and incubated at room temperature for 15 minutes. The signal was measured using the VICTOR™ X3 Multilabel Plate Reader with an excitation wavelength of 485–500 nm and emission filter of 520–530 nm. Data were normalized to the untreated control.

### 2.9. Cell Viability by Annexin V/PI Flow Cytometric Assay

Cell viability was measured by flow cytometry on cells cultured for 96 h in normal growth medium with different concentrations of DMOG (0, 0.01, 0.1, and 1 mM) using Annexin V-FITC Apoptosis Detection Kit (eBioscience), according to the manufacturer's protocol.

Briefly, adherent cells were trypsinized, washed in PBS by gentle shaking, and resuspended at the concentration of 5∗10^5^ cells/mL with 200 *μ*L of a specific binding buffer (10 mM HEPES/NaOH, pH 7.4; 140 mM NaCl, and 2.5 mM CaCl_2_) containing 5 *μ*L of Annexin V-FITC. After incubation for 10 min in the dark at room temperature, cells were washed in PBS, resuspended in 190 *μ*L of binding buffer, and then stained with 10 *μ*L propidium iodide (20 *μ*g/mL). Samples were immediately acquired with a Navios Flow Cytometer (Beckman Coulter) and analyzed using Kaluza 1.2 software (Beckman Coulter).

### 2.10. RNA Extraction and Real-Time PCR

Total RNA was isolated using TRIzol® Reagent (Ambion, Life Technologies), and 1 *μ*g of extracted RNA was reverse transcribed to cDNA using the iScript cDNA synthesis kit (BioRad), according to the manufacturer's instructions. Real-time PCR was performed in a 96-well plate with 10 ng of cDNA as a template, 0.2 *μ*M primers, and 1x Power SYBR® Green PCR Master Mix (Applied Biosystems, Life Technologies) in a 20 *μ*L final volume per well using a StepOnePlus™ Real-Time PCR System (Applied Biosystems). The mRNA levels of vascular endothelial growth factor (VEGF), Nanog homeobox (NANOG), octamer-binding transcription factor 4 (OCT4), Kruppel-like factor 4 (KLF4), Tenascin C, collagen type I alpha-1 (COL1A1), peroxisome proliferator-activated receptor-*γ* (PPAR-*γ*), lipoprotein lipase (LPL), human alkaline phosphatase (ALP), and SRY-box9 (SOX9) were assessed. Ribosomal protein S14 (S14) was used as the housekeeping gene in all quantitative analyses (Table 1).

Amplification protocol: initial denaturation at 95°C for 3 minutes followed by 40 cycles of 5 seconds each at 95°C and 30 seconds at 57°C. Relative quantification of target genes was performed in triplicates, analyzed using the 2^−ΔΔCt^ method and normalized to the corresponding S14 values.

### 2.11. Adipogenic Differentiation

Cells were plated at a concentration of 3 × 10^4^ cells/cm^2^ and cultured in DMEM-low glucose (Sigma-Aldrich), 10% FBS (HyClone, Thermo Fisher Scientific), 4 mM L-glutamine (Euroclone), and 1% antibiotic-antimycotic mixture (Euroclone), with the addition of the mesenchymal stem cell adipogenesis kit (Millipore) for 21 days, according to the manufacturer's instructions. The adipogenic medium was changed every other day. At day 21, Oil Red O solution (Millipore) was used to stain lipid droplets of derived adipocytes, according to the manufacturer's procedures. All photomicrographs were acquired with an Axiovert 40 microscope (Zeiss) equipped with a Moticam 2300 camera (Motic). The mRNA expression of adipogenic markers including PPAR-*γ* and LPL were also assessed on days 7 and 21 by real-time PCR, as described above [17].

### 2.12. Osteogenic Differentiation

Cells were plated at the concentration of 3 × 10^4^ cells/cm^2^ and cultured in the osteogenesis induction medium, which was constituted of DMEM-low glucose (Sigma-Aldrich), 10% FBS (HyClone, Thermo Fisher Scientific), 4 mM L-glutamine (Euroclone), and 1% antibiotic-antimycotic mixture (Euroclone), supplemented with 0.1 *μ*M dexamethasone, 50 *μ*g/mL L-ascorbic acid-2-phosphate, and 10 mM *β*-glycerophosphate (all reagents from Sigma-Aldrich) for 17 days. The osteogenic medium was changed every other day. At day 17, Alizarin Red solution (Millipore) was used to detect calcium deposition in derived osteoblasts, according to the manufacturer's instruction. All photomicrographs were acquired with an Axiovert 40 microscope (Zeiss) equipped with a Moticam 2300 camera (Motic). The expression of osteogenic marker ALP was determined by real-time PCR on days 5 and 17, as described above [17].

### 2.13. Chondrogenic Differentiation

Chondrogenic differentiation was performed using StemPro Chondrogenesis Differentiation Kit (Life Technologies), according to the manufacturer's instruction. Briefly, cells were resuspended in growth medium at the concentration of 1.6 × 10^7^, and 5 *μ*L droplets were seeded in the center of a multiwell plate wells. After two hours, the chondrogenesis induction medium provided by the kit was added. The chondrogenic medium was changed every 2-3 days for 14 days. Alcian blue solution (Bio-Optica) was used to stain acid mucopolysaccharides formed during the differentiation process. The expression of chondrogenic marker SOX9 was determined by real-time PCR on days 5 and 14, as described above [17].

### 2.14. Statistical Analysis

Statistical analysis was performed using GraphPad Prism v 6.0 software (GraphPad Software Inc.). Data were typical results from three replicate experiments for each of the six patient-derived cell lines and were expressed as the mean ± standard deviation (SD). The Shapiro-Wilk normality test was used to evaluate the normal distribution of the sample.

One-way analysis of variance (ANOVA), followed by two-tailed, paired Student's *t*-test or Mann–Whitney test according to the characteristics of the data distribution, wherever applicable, was used for multiple comparisons. Linear contrasts were used to assess for changes in the mean values with increasing doses of DMOG. The significance level was set at *p* value lower than 0.05.

## 3. Results

### 3.1. Effects of DMOG on HIF-1*α* Stabilization on hTSCs

**Figure 2:**
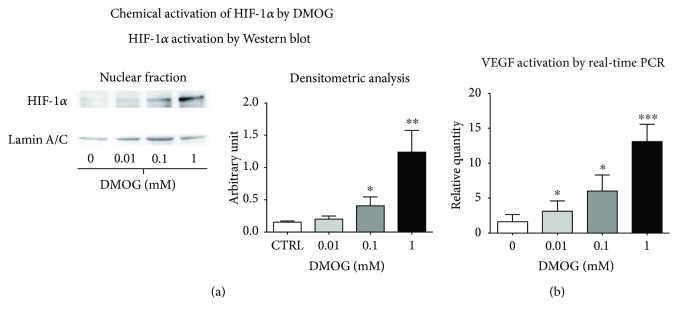
HIF-1*α* chemical activation with DMOG. HIF-1*α* expression was analyzed by Western blot (a). The activation of HIF-1*α* was also analyzed measuring the expression of VEGF by real-time PCR (b). Western blots were quantified by densitometry and results expressed with arbitrary units. Real-time PCR data are expressed as fold change as compared to control cells without DMOG treatment. *p* values were calculated using Student's *t*-test. Only *p* values < 0.05 are indicated: ^∗^ *p* < 0.05, ^∗∗^ *p* < 0.01, and ^∗∗∗^ *p* < 0.001.

**Figure 3:**
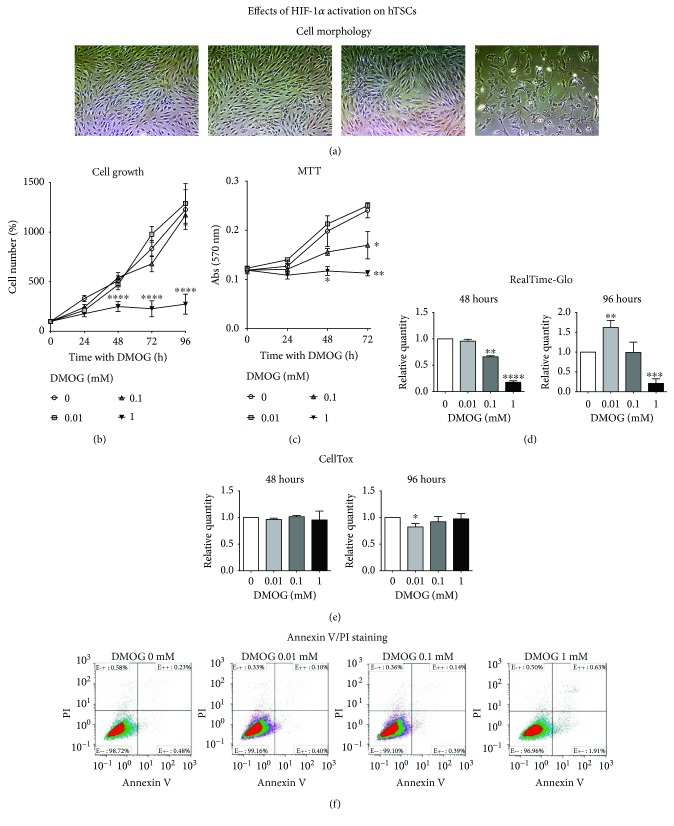
Effects of HIF-1*α* activation on hTSC morphology, proliferation, and viability. (a) Phase-contrast microphotographs (original magnification ×10), (b) cell growth curves, (c) MTT assay, (d) RealTime-Glo assay, (e) CellTox assay, and (f) Annexin V/PI staining of hTSCs during a 96 h treatment with different concentrations of DMOG (0.01 mM, 0.1 mM, and 1 mM) in normal growth medium and compared to control cells cultured without DMOG (0 mM). All experiments were performed in triplicates. Error bars show the mean ± SD of six and three different experiments in the case of cell growth curves and MTT assay, respectively. *p* values were calculated using Student's *t*-test. Only *p* values < 0.05 are indicated: ^∗^ *p* < 0.05, ^∗∗^ *p* < 0.01, ^∗∗∗^ *p* < 0.001, and ^∗∗∗∗^ *p* < 0.0001, as compared to control cells. Bar = 100 *μ*m.

To chemically activate HIF-1*α*, hTSCs were cultured under normoxia in the presence of DMOG at different concentrations (0.01 mM, 0.1 mM, and 1 mM) for 96 h and compared to control cells that were cultured in normal growth medium without DMOG (Figure 2). Analysis of HIF-1*α* nuclear localization by Western blot revealed that the factor was activated by DMOG in a dose-dependent manner (Figure 2(a)). Similarly, analysis of VEGF, one of the main HIF-1*α* target genes, showed an expression increase by real-time PCR that was proportional to the concentration of DMOG in the culture medium (Figure 2(b)). Cell morphology analysis by phase-contrast microscopy revealed no noticeable differences between control and DMOG-treated hTSCs (Figure 3(a), 96 h of DMOG treatment). However, DMOG at 1 mM concentration induced cell suffering, as the formation of vacuoles and cell proliferation arrest could be observed throughout the culture plate (Figure 3(a)). The evaluation of the effects of DMOG on cell proliferation revealed a slight, although not statistically significant, increase when cells were treated with 0.01 mM DMOG (Figure 3(b)). On the other hand, a dose-dependent reduction could be initially observed at 0.1 mM after 72 h of treatment, reaching a significant decrease of 51%, 72.6%, and 77.7% in the presence of 1 mM DMOG at 48, 72, and 96 h, respectively, as compared to control cells (Figure 3(b)). These results were confirmed by MTT cell metabolic activity assay where a DMOG dose-dependent reduction could be observed at all time points (24, 48, and 72 h), reaching a 53.1% reduction at 1 mM, as compared to control cells (Figure 3(c)). Similarly, RealTime-Glo™ cell viability tests showed a DMOG dose-dependent signal reduction, with the exception of 0.01 mM DMOG that showed a significant increase at 96 h as compared to control cells (Figure 3(d)). Cell toxicity tests revealed no significant differences at all DMOG concentrations at 48 and 96 h, with the exception of 0.01 mM DMOG that showed a minor, yet statistically significant, decrease in cell toxicity at 96 h. Cell death analysis by Annexin V test revealed no significant cell apoptosis at all tested DMOG concentrations (Figure 3(f)).

### 3.2. Effects of HIF-1*α* Stabilization on hTSC Stemness

**Figure 4:**
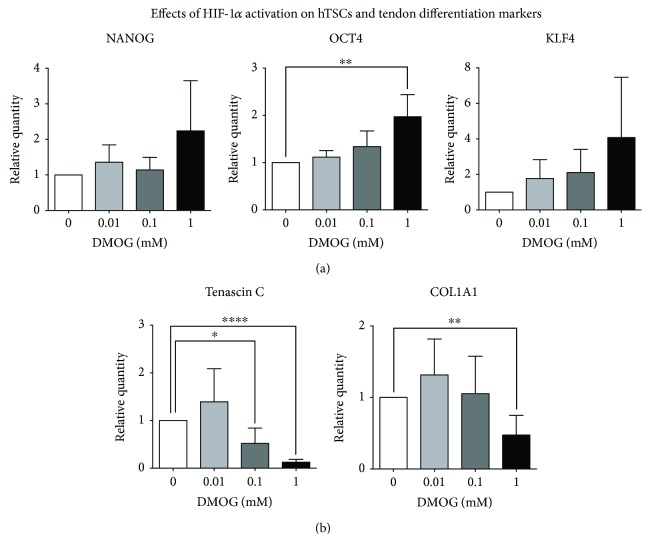
Effects of HIF-1*α* activation on stem cell and tendon marker expression. (a) Gene expression of NANOG, OCT4, and KLF4 by real-time PCR after a 96 h DMOG treatment, as compared to control-untreated cells. (b) Gene expression of Tenascin C and COL1A1 by real-time PCR after a 96 h DMOG treatment, as compared to control-untreated cells. Values are expressed as fold changes relative to control cells. Data are expressed as means ± SD of six different experiments. One-way ANOVA was used to test for differences among DMOG-treated cell groups. Bonferroni post hoc test was performed to compare individual DMOG-treated group cells against each other to see where the significant differences lie. Linear contrast analysis (analysis of variance) was used to assess for changes in the mean values with increasing doses of DMOG (red lines). Only *p* values < 0.05 are indicated: ^∗^ *p* < 0.05, ^∗∗^ *p* < 0.01, and ^∗∗∗∗^ *p* < 0.0001, as compared to control cells.

To evaluate the effects of DMOG on hTSC stemness, the expression of stem cell markers NANOG, OCT4, and KLF4 was determined by real-time PCR after a 96 h DMOG treatment at 0.01 mM, 0.1 mM, and 1 mM and compared to those of control cells (Figure 4(a)). The results showed a statistically significant trend increase of the mRNA levels of NANOG, OCT4, and KLF4, as compared to control cells, and that it was proportional to the concentration of DMOG used (red tendency line, Figure 4(a)). The mRNA levels of Tenascin C and COL1A1 tendon markers were measured by real-time PCR in hTSCs exposed to different concentrations (0.01 mM, 0.1 mM, and 1 mM) of DMOG for 96 h. The results showed a statistically significant trend decrease of the mRNA levels of both tendon markers, which was proportional to the concentration of DMOG used (red tendency line, Figure 4(b)). In particular, the expression of both Tenascin C and COL1A1 showed a slight, although not significant, increase when cells were treated with 0.01 mM DMOG, as compared to control cells. On the other hand, a significant reduction, which was concentration dependent, could be observed at 0.1 mM and 1 mM DMOG, reaching an 8.1- and 2.1-fold decrease for Tenascin C and COL1A1, respectively, at 1 mM DMOG concentration (Figure 4(b)).

### 3.3. Effects of HIF-1*α* Stabilization on hTSC Differentiation

**Figure 5:**
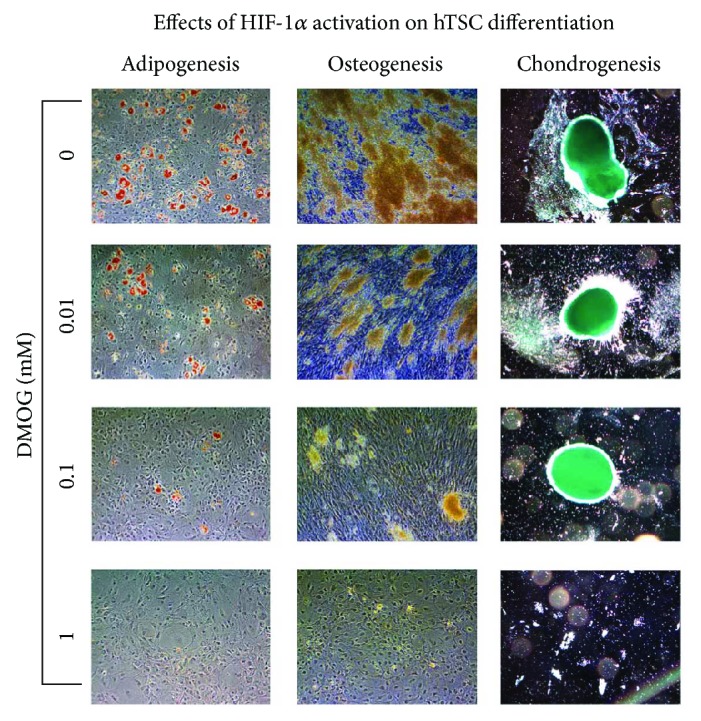
Effects of HIF-1*α* activation on in vitro differentiation of hTSCs toward the adipogenic, osteogenic, and chondrogenic phenotypes. Adipogenic, osteogenic, and chondrogenic differentiation ability of hTSCs in the presence of DMOG at different concentrations (0 mM, 0.01 mM, 0.1 mM, and 1 mM) in the appropriate differentiation medium was qualitatively evaluated by (a) Oil Red O, (b) Alizarin Red S staining, and (c) Alcian blue staining, respectively. (a) Lipid intracellular droplets (red) in the adipocytes were stained with Oil Red O solution. (b) Alizarin Red S staining revealed the presence of calcium deposits (yellowish-brown). (c) Alcian blue staining was used to stain acid mucopolysaccharides formed during the differentiation process. Typical results are shown. Original magnification ×10. Bar = 100 *μ*m.

**Figure 6:**
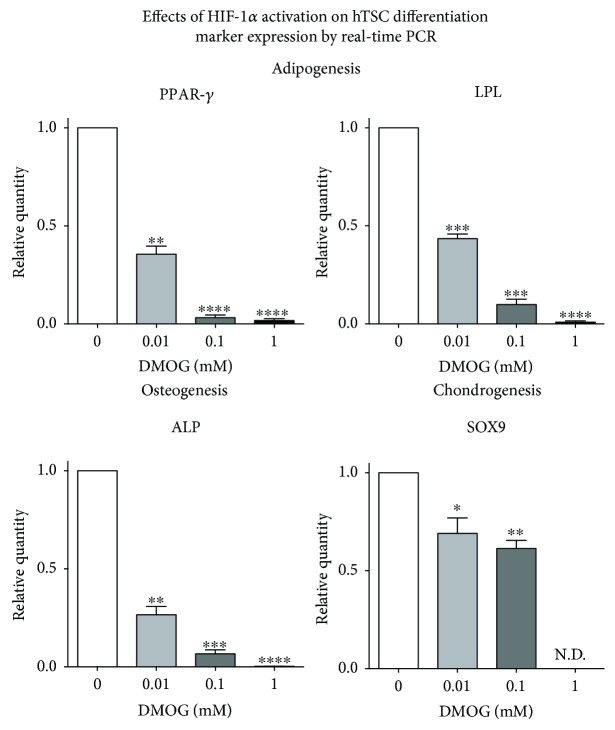
Quantitative evaluation of the effects of HIF-1*α* activation on in vitro differentiation of hTSCs toward the adipogenic, osteogenic, and chondrogenic phenotypes. Peroxisome proliferator-activated receptor-*γ* (PPAR-*γ*), lipoprotein lipase (LPL), and human alkaline phosphatase (ALP) expression by real-time PCR in hTSCs induced to differentiate toward adipocytes, osteoblasts, and chondroblasts in the presence of different concentrations of DMOG (0.01 mM, 0.1 mM, and 1 mM). Control cells were cultured under the same conditions but without DMOG. Values are expressed as fold changes relative to control cells. Data are expressed as the mean ± SD of three different experiments. *p* values were calculated using Student's *t*-test. Only *p* values < 0.05 are indicated: ^∗^ *p* < 0.05, ^∗∗^ *p* < 0.01, ^∗∗∗^ *p* < 0.001, and ^∗∗∗∗^ *p* < 0.0001, as compared to control cells.

To assess the effects of HIF-1*α* activation on the multipotency of hTSCs, cells were induced to differentiate toward osteoblasts, adipocytes, and chondroblasts by culturing them in the appropriate differentiation medium for 17, 21, and 28 days, respectively, in the presence of DMOG at different concentrations (0, 0.01, 0.1, and 1 mM), as described in the Methods (Figure 5). The adipogenic differentiation was qualitatively evaluated by Oil Red O staining revealing a reduction in the amount of lipid intracellular droplets, which was proportional to the amount of DMOG in the culture medium (Figure 5, left column). The osteogenic differentiation was assessed qualitatively by Alizarin Red S staining, which showed that the amount of calcium deposits was markedly reduced, in a dose-dependent manner, when cells were cultured in the presence of DMOG, as compared to control cells (Figure 5, central column). The chondrogenic differentiation was assessed qualitatively by Alcian blue staining, showing a deposition of extracellular matrix proteins when cells were cultured in the presence of DMOG, especially at 1 mM, as compared to control cells (Figure 5, right column). The effects of HIF-1*α* activation on adipogenesis and osteogenesis were also analyzed quantitatively by determining the mRNA levels of adipogenic markers PPAR-*γ* and LPL, osteogenic marker ALP, and chondrogenic marker SOX9 by real-time PCR (Figure 6). The results showed a significant decrease of all markers that was proportional to the concentration of DMOG used (Figure 6). In particular, PPAR-*γ* showed 2.8-, 31.7-, and 59.7-fold expression decrease, while LPL showed 2.3-, 10.1-, and 109.2-fold decrease for 0.01 mM, 0.1 mM, and 1 mM DMOG treatment, as compared to control cells, respectively (Figure 6). Osteogenic differentiation in the presence of DMOG was inhibited, as revealed by a significant ALP decrease that was proportional to the concentration of DMOG used (Figure 6). In particular, ALP showed 3.7-, 15.1-, and 386-fold decrease when cells were incubated with 0.01 mM, 0.1 mM, and 1 mM DMOG, respectively, as compared to control cells (Figure 6).

**Figure 7:**
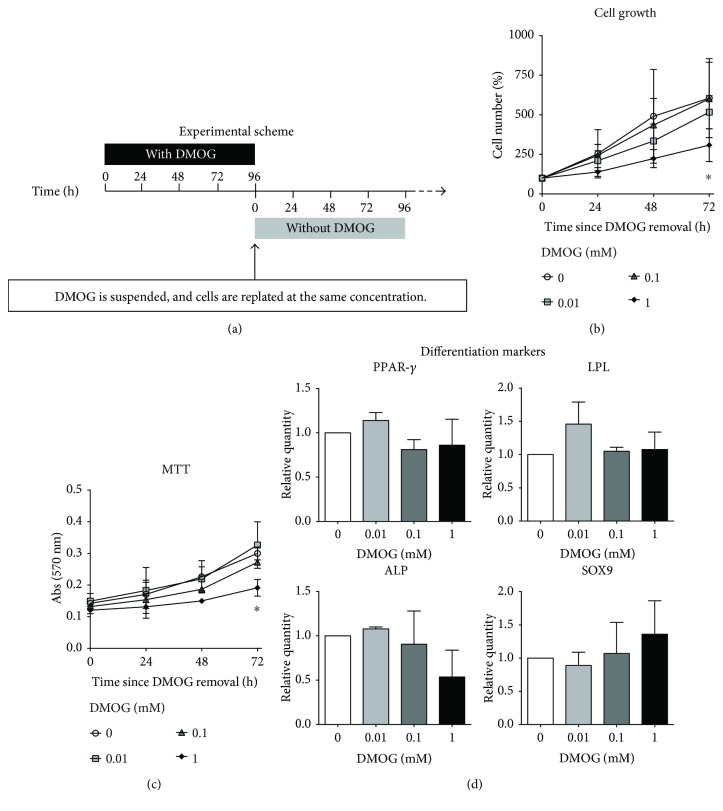
Reversibility of HIF-1*α* activation effects on hTSC proliferation, viability, and differentiation markers. (a) Schematic representation of the experimental setup. hTSCs were cultured in the presence of different concentrations of DMOG (0 mM, 0.01 mM, 0.1 mM, and 1 mM) in normal growth medium for 96 h, washed with PBS, detached, replaced at the same concentration in normal growth medium without DMOG, and then left overnight before successive analyses at 24, 48, 72, and 96 h after seeding. (b) Cell growth curves and (c) MTT assay of hTSCs after culturing DMOG-pretreated cells for 96 h in DMOG-free medium normal and compared to control cells (0 mM DMOG). (d) Peroxisome proliferator-activated receptor-*γ* (PPAR-*γ*), lipoprotein lipase (LPL), and human alkaline phosphatase (ALP) expression by real-time PCR in hTSCs induced to differentiate toward adipocytes, osteoblasts, and chondroblasts after a 96 h preconditioning with DMOG, followed by 96 h in normal DMOG-free growth medium, and then differentiation in DMOG-free medium. Control cells were cultured under the same conditions but without DMOG. Values are expressed as fold changes relative to control cells. All experiments were performed in triplicates. Data are expressed as the mean ± SD of six in the case of cell growth curves and three different experiments in case of MTT assay and differentiation marker expression. *p* values were calculated using Student's *t*-test. Only *p* values < 0.05 are indicated: ^∗^ *p* < 0.05, as compared to control cells.

Finally, it was tested whether the effects of HIF-1*α* activation could be reverted. To this purpose, hTSCs were grown in the presence of 0.01 mM, 0.1 mM, and 1 mM DMOG for 96 h, harvested, replated at the same cell concentration, and then cultured in normal growth medium (Figure 7(a)). Cells were then harvested and counted every 24 h for 4 days, starting 24 h after seeding. Cell growth analyses revealed that cells pretreated with 0.01 mM and 0.1 mM DMOG could regain the normal proliferation capacity, analogous to control cells, when switched back to the normal growth medium (Figure 7(b)). On the other hand, cells treated with 1 mM DMOG did not completely recover from DMOG treatment, at least within the experiment timeframe, as they showed a markedly proliferation reduction at 72 h, reaching 58.4% decrease at 96 h, as compared to control cells (Figure 7(b)). MTT assays confirmed cell growth experiments, revealing that DMOG treatment effects could be reverted within 72 h after switching cells in normal growth medium, with the exception of 1 mM DMOG, that still showed a significant reduction (about 36.2% at 72 h), as compared to control cells (Figure 7(c)). Then, the reversibility of the effects of HIF-1*α* activation on cell differentiation was determined. To this purpose, cells were first pretreated for 96 h with DMOG, followed by 96 h in normal growth medium to reestablish normal proliferation, and then differentiated in DMOG-free medium. Analysis of the adipogenic, osteogenic, and chondrogenic differentiation markers revealed no significant difference between DMOG pretreated and control cells at all concentrations (*p* > 0.05) (Figures 7(c) and 7(d)).

## 4. Discussion

Adult stem cells have been found to reside in hypoxic tissue compartments often referred to as “stem cell niches.” Although the microenvironment and the molecular signals present in the niches have been only partially elucidated, it is now accepted that a low-oxygen tension helps to maintain the undifferentiated state of the progenitor cells [18, 19]. Moreover, it has been shown that culturing stem cells in vitro in low-oxygen incubators mimics the “niche,” at least partially and cells seem to maintain their potency without differentiating [18, 20]. However, as already reported in the Introduction, the effects of low oxygen on adult stem cells appear to be very variable and cell subtype dependent, and it has been also reported that it can reduce stem cell proliferation and favor differentiation. Clearly, finding a way of amplifying stem cells without compromising their potency is vital for reaching the cell numbers needed for their therapeutic application. Along this line, the use of hypoxic incubators has been shown to be feasible, and the possibility of pharmacologically mimic oxygen deprivation represents a simpler and cost-effective alternative. Chemically mimicking the hypoxic niche has several advantages over culturing cells in low oxygen: (a) a drug can be easily synthesized on a large scale, (b) the methodology does not require especially designed and expensive incubators, (c) the drug dosage can be easily adjusted and controlled, and (d) the treatment can be suspended at any time. On the other hand, controlling the oxygen tension can be very cumbersome, especially when cells are cultured for a prolonged time and the media needs to be replaced without exposing cells to normal-oxygen tension. This can be achieved only with specially designed and expensive culturing chambers, which are not commonly used in most laboratories or research hospitals. Actually, most studies under hypoxic conditions are performed with normal incubators connected to a nitrogen tank; thus, cells are constantly exposed to cycles of low/high oxygen. This unfortunately mimics the ischemia/reperfusion conditions that might be detrimental for maintaining stem cells, as they could stimulate their differentiation, as it happens, for instance, after a cardiac infarct [21].

On these bases, with so many conflicting results, it is very hard to come up with a general picture. However, it is clear that under oxygen deprivation, any stem cell activates a common response mechanism, which is mainly regulated by the hypoxia-inducible factor. In particular, HIF-1*α*, which is activated under low-oxygen tension, has been shown to play a pivotal role in stem cell differentiation [11, 18]. In particular, the activation of HIF-1*α* has been shown to inhibit stem cell differentiation [3, 22]. Moreover, activation of HIF-1*α* is known to enhance the cell defense machinery against apoptosis [23–26]. This is another crucial issue in stem cell therapy, as most injected cells usually die because they have to survive in a very hostile environment while trying to reach the damaged tissue. Indeed, preconditioning stem cells by activating HIF-1*α* has been shown to increase their survival after injection and ultimately obtain better regenerative results [27, 28]. Thus, in this study, we tested the effects of HIF-1*α* activation on human adult tendon stem cells, which we recently isolated from the rotator cuff supraspinatus tendon [15]. This tissue is quite hypoxic, as it is poorly vascularized in the adults, and this could explain, at least partially, its limited healing capacity. To have a better control on HIF-1*α* activation, we decided to modulate it with a pharmacological approach using DMOG at different concentrations, which allowed to easily fine-tune HIF-1*α* expression. As we anticipated, in this study, we found that tendon stem cells cultured in the presence of DMOG reduce their proliferation capability and the effect is proportional to the concentration used. This is in agreement with other reports on adult stem cells cultured under hypoxia [29, 30]. Actually, a tendency, although not significant, toward a proliferation increase could be observed at very low DMOG concentration (0.01 mM), which is also in agreement with other literature reports, showing that moderate hypoxia can stimulate stem cell proliferation, while extreme hypoxia induces them to exit the cell cycle and enter a quiescent state [9]. Nonetheless, we found that DMOG effects on cell proliferation are reversible, as cells could regain their proliferation capability once the molecule is removed from the culture medium. We also found that stem cell markers NANOG, OCT4, and KLF4 are slightly upregulated upon DMOG supplementation and this increase is proportional to the DMOG concentration used. Moreover, tendon-specific markers decrease during DMOG treatment, supporting the notion that chemically induced hypoxia can keep stem cells into a more undifferentiated state. Furthermore, DMOG effects on tendon stem cell differentiation toward osteoblasts and adipocytes were also assessed. The results showed that differentiation is hindered when HIF-1*α* is activated in the presence of DMOG and that the inhibition is proportional to the concentration of DMOG used, supporting the notion that stem cells reside in the tissue in hypoxic niches, where HIF-1*α* is activated and the differentiation is inhibited, until cells migrate to more oxygenated areas. Actually, the literature on this issue is quite controversial. In fact, while some reports show a direct increase in the differentiation markers upon in vitro differentiation of hypoxia-activated mesenchymal stem cells [29, 31–34], other reports showed a marked reduction of differentiation [3, 18, 22, 30, 35–40]. Indeed, while the general notion is that a more primitive stem cell should give better regenerative results, it could be argued that a more committed stem cell is more prone to differentiate than an immature one, especially when different stem cell progenitors are compared in the same differentiation timeframe. In fact, at this stage, we cannot exclude that DMOG pretreated cells, as they show a higher expression of stem cell markers, could be more effective in regenerating damaged tissue in vivo.

## 5. Conclusions

In conclusion, in this study, we found that a chemical activation of HIF-1*α* reduces human tendon stem cell proliferation, increases the expression of stem cell markers, and reduces their commitment toward the tendon phenotype. These effects are reversible upon DMOG removal from the culture medium. Moreover, DMOG present in the differentiation medium reversibly inhibits stem cell differentiation. Further studies to test whether DMOG pretreatment has any beneficial effects in vivo in tendon stem cell regeneration capacity are currently undergoing in our laboratories.

## Figures and Tables

**Table 1:** 

Gene	Primer sequence
S14	Fw: 5′-GTGTGACTGGTGGGATGAAGG-3′
	Rev: 5′-TTGATGTGTAGGGCGGTGATAC-3′

VEGF	Fw: 5′-CAACATCACCATGCAGATTATGC-3′
	Rev: 5′-TCGGCTTGTCACATTTTTCTTGT-3′

NANOG	Fw: 5′-GGTCCCAGTCAAGAAACAGA-3′
	Rev: 5′-GAGGTTCAGGATGTTGGAGA-3′

OCT4	Fw: 5′-AGGAGAAGCTGGAGCAAAA-3′
	Rev: 5′-GGTCGAATACCTTCCCAAA-3′

KLF4	Fw: 5′-GACTTCCCCCAGTGCTTC-3′
	Rev: 5′-CGTTGAACTCCTCGGTCTC-3′

Tenascin C	Fw: 5′-CGGGGCTATAGAACACCAGT-3′
	Rev: 5′-AACATTTAAGTTTCCAATTTCAGGTT-3′

COL1A1	Fw: 5′-GGGATTCCCTGGACCTAAAG-3′
	Rev: 5′-GGAACACCTCGCTCTCCA-3′

PPAR-*γ*	Fw: 5′-TTCCTTCACTGATACACTGTCTGC-3′
	Rev: 5′-GGAGTGGGAGTGGTCTTCCATTAC-3′

LPL	Fw: 5′-AGAGAGAACCAGACTCCAATG-3′
	Rev: 5′-GGCTCCAAGGCTGTATCC-3′

ALP	Fw: 5′-CGCACGGAACTCCTGACC-3′
	Rev: 5′-GCCACCACCACCATCTCG-3′

SOX9	Fw: 5′-GTACCCGCACTTGCACAAC-3′
	Rev: 5′-TCGCTCTCGTTCAGAAGTCTC-3′

## Abbreviations

ALP: Alkaline phosphatase
COL1A1: Collagen type I alpha-1
DMOG: Dimethyloxalylglycine
HIF-1*α*: Hypoxia inducible factor-1*α*
KLF4: Kruppel-like factor 4
LPL: Lipoprotein lipase
NANOG: Nanog homeobox
OCT4: Octamer-binding transcription factor 4
PHDs: HIF prolyl hydroxylases
PPAR-*γ*: Peroxisome proliferator-activated receptor-*γ*
S14: Ribosomal protein S14
SOX9: SRY-box9
VEGF: Vascular endothelial growth factor.

## Supplementary Materials

Supplementary Materials Supplementary Figure 1: characterization of hTSCs by flow cytometry. Flow cytometric analysis of antigens CD34, CD45, HLA-DR, CD73, CD90, and CD105. Peaks of specific antigens are shown in green while peaks of respective isotype controls are shown in red.
