# Supplementary figures and images for: Chemical Activation of the Hypoxia-Inducible Factor Reversibly Reduces Tendon Stem Cell Proliferation, Inhibits Their Differentiation, and Maintains Cell Undifferentiation
Source: Stem Cells Int. 2018 Mar 11;2018:9468085. doi: 10.1155/2018/9468085 (PMC5866868; doi:10.1155/2018/9468085)

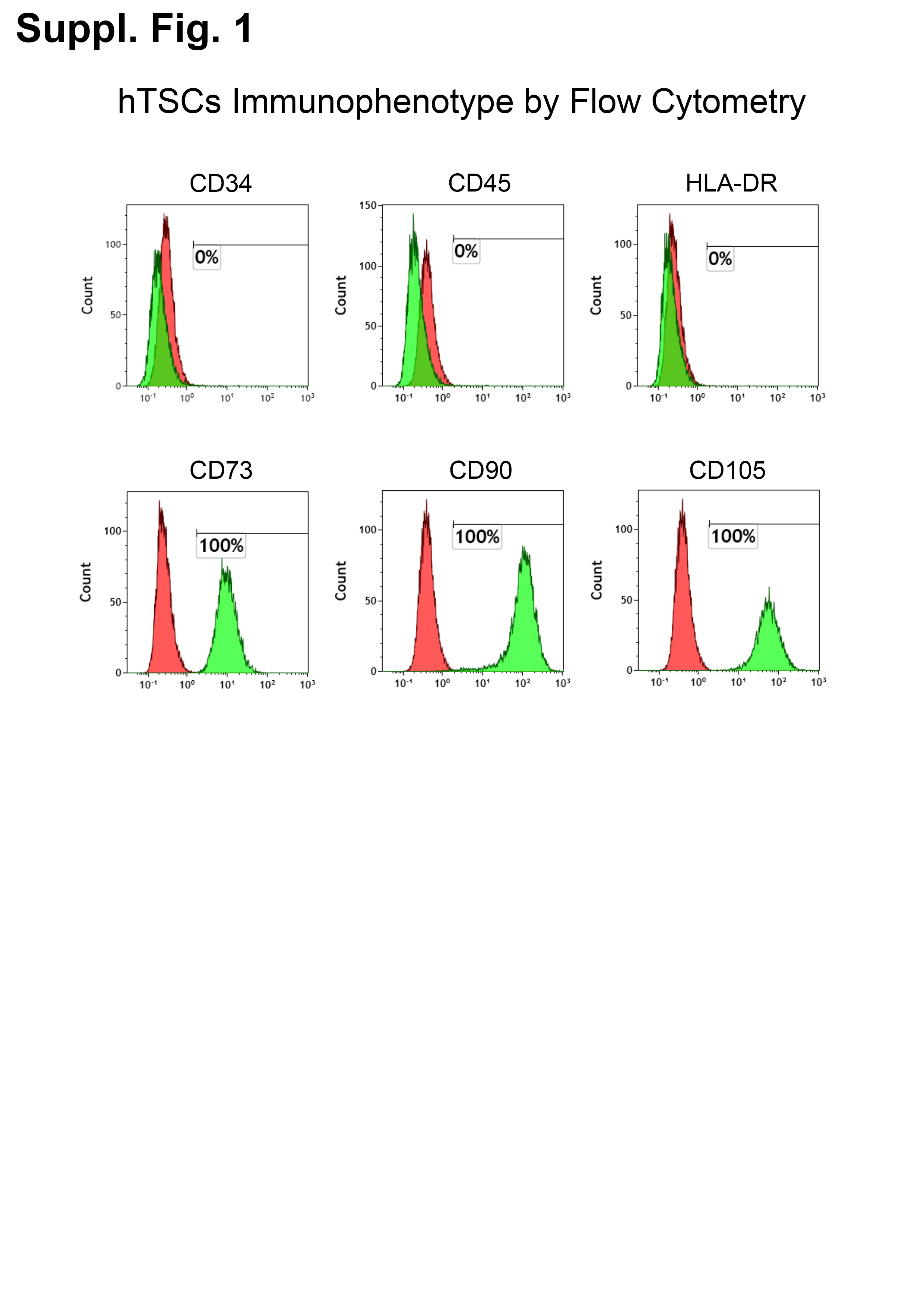

Supplement: Supplementary Materials — Supplementary Figure 1: characterization of hTSCs by flow cytometry. Flow cytometric analysis of antigens CD34, CD45, HLA-DR, CD73, CD90, and CD105. Peaks of specific antigens are shown in green while peaks of respective isotype controls are shown in red. [file 9468085.f1.docx]
